# Prediction of coating thickness for polyelectrolyte multilayers via machine learning

**DOI:** 10.1038/s41598-021-98170-x

**Published:** 2021-09-21

**Authors:** Varvara Gribova, Anastasiia Navalikhina, Oleksandr Lysenko, Cynthia Calligaro, Eloïse Lebaudy, Lucie Deiber, Bernard Senger, Philippe Lavalle, Nihal Engin Vrana

**Affiliations:** 1 Inserm UMR_S 1121, Biomaterials and Bioengineering, Centre de Recherche en Biomédecine de Strasbourg, 67000 Strasbourg, France; 2 Université de Strasbourg, Faculté de Chirurgie Dentaire, 67000 Strasbourg, France; 3 SPARTHA Medical, 67100 Strasbourg, France; 4 PRESTE, 75011 Paris, France

**Keywords:** Biomaterials, Nanobiotechnology

## Abstract

Layer-by-layer (LbL) deposition method of polyelectrolytes is a versatile way of developing functional nanoscale coatings. Even though the mechanisms of LbL film development are well-established, currently there are no predictive models that can link film components with their final properties. The current health crisis has shown the importance of accelerated development of biomedical solutions such as antiviral coatings, and the implementation of machine learning methodologies for coating development can enable achieving this. In this work, using literature data and newly generated experimental results, we first analyzed the relative impact of 23 coating parameters on the coating thickness. Next, a predictive model has been developed using aforementioned parameters and molecular descriptors of polymers from the DeepChem library. Model performance was limited because of insufficient number of data points in the training set, due to the scarce availability of data in the literature. Despite this limitation, we demonstrate, for the first time, utilization of machine learning for prediction of LbL coating properties. It can decrease the time necessary to obtain functional coating with desired properties, as well as decrease experimental costs and enable the fast first response to crisis situations (such as pandemics) where coatings can positively contribute. Besides coating thickness, which was selected as an output value in this study, machine learning approach can be potentially used to predict functional properties of multilayer coatings, e.g. biocompatibility, cell adhesive, antibacterial, antiviral or anti-inflammatory properties.

## Introduction

Layer-by-layer (LbL) coating is a method for surface modification based on the electrostatic interactions between two polyelectrolytes^1,2^. Such coating is developed thanks to successive deposition of polycations and polyanions onto the surface of a material, and by performing a rinsing step after each deposition. This method is very versatile as a large number of polyelectrolytes can be used, making it possible to adapt the coating for a particular application. Different methods can be used for the build-up of LbL coatings, such as dip-coating, spin-coating, and spraying^3,4^. The most used method and perhaps the easiest one is dip-coating, but it is also more time-consuming compared to spin-coating for instance^5^.

LbL coatings are used for multiple biomedical applications, in particular, because natural polyelectrolytes presenting good biocompatibility can be used for LbL film build-up. It is possible to develop antibacterial surfaces, smart healing materials, and coatings for tissue engineering. Moreover, LbL coatings can be used for loading drugs or other bioactive molecules, which allows their local delivery^6–9^. Non-biomedical LbL applications include construction of gas barrier films^10^, optical fiber sensing^11^, and many electrochemical systems^12^.

However, the empirical manner of polycation/polyanion selection is an impediment on rapid new coating development. First, the formation of the coatings can be very long, if many layers are required, and for thick films, the method can become fastidious. Secondly, the thickness of the different coatings is difficult to control, as it depends on different parameters such as temperature, pH, ionic strength, and others^5^.

Moreover, there remain difficulties in understanding how interactions between polymers occur, as they are mostly multifactorial. Thus, LbL coatings growth can be different (in most cases linear or exponential, at least up to a given number of layers deposited) depending on polymers’ properties and on diffusion between layers^13^. Experimentally, different methods are used to evaluate LbL film thickness: quartz crystal microbalance with dissipation monitoring (QCM-D) can be used to follow step-by-step polyelectrolyte deposition with high accuracy. Other methods such as atomic force microscopy (AFM), confocal microscopy or ellipsometry can also be used^14,15^. However, these different methods do not use the same approach of thickness determination, and for the same LbL film, the results obtained by different techniques can differ.

As a result, and despite the progress made in the field, the data accumulated over the years do not provide predictive capacities on how a given couple of polymers will form an LbL film, which also decreases the rate of advance in the field. In this work, we hypothesize that using the current state-of-the-art data science techniques, we can determine how different parameters affect coating thickness and predict the thickness of the new coatings. To do so, we used historical and generated data for predictive model development using machine learning.

Machine learning is an approach which uses algorithms that improve upon training on large datasets and is able to find complex patterns, make predictions and decisions. In this work, we used two training datasets: one comprising the data extracted from literature (several thickness determination methods) and another containing experimental data produced in our laboratory (thickness determination by QCM-D).

**Figure 1:**
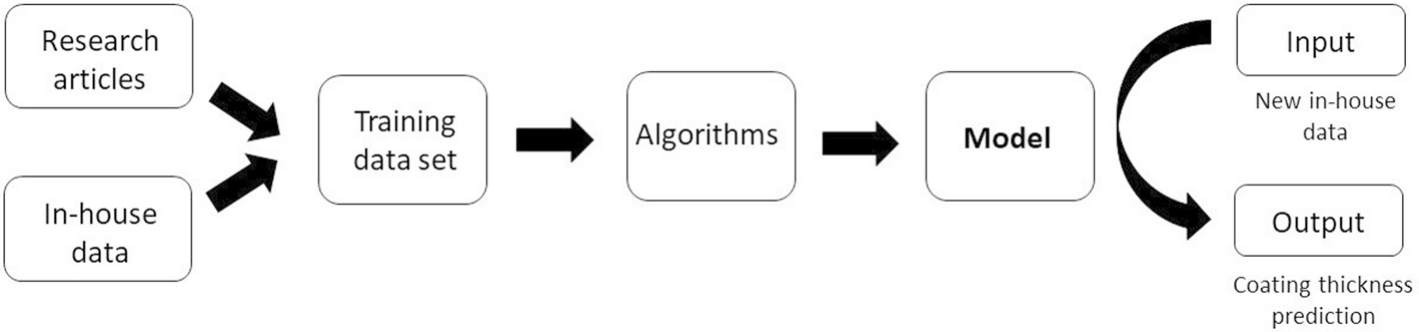
Workflow for coating thickness prediction using supervised machine learning approach.

In the first part of the work, the most important parameters influencing coating buildup were determined. In the second part, a thickness predictive model was built using the training set, and its performance was evaluated. Finally, we validated the model on LbLs which were not in the training set, and were able to predict the coating thickness (Fig. 1). To our knowledge, this is the first time that machine learning approach has been used for LbL coating thickness prediction.

## Results and discussion

### Data collection

The first step of the work consisted in data collection from the literature, which represents the first dataset. It should be stated that currently in the literature no established database is available related to LbL coating thickness, and the available experimental data is relatively scarce compared to the number of LbL related articles. The second dataset was based on the QCM-D experiments done in the laboratory. For the data extracted from the literature, different ways of thickness calculation/estimation were used, such as AFM, ellipsometry, confocal microscopy. Coating thickness determined by these methods may differ, but due to the limited amount of the available data, we selected to include all the data regardless of the thickness measurement method. All the results extracted from the literature, as well as obtained in the laboratory, were entered in the tables describing different parameters (Table S1). Of note, all the multilayers used in the study were prepared by dip-coating or a similar technique (simple polymer solution deposition on the substrate followed by adsorption time and rinsing). Thus, the coating preparation method was not among the parameters influencing thickness.

### Influence of different construction parameters on coating thickness

**Figure 2:**
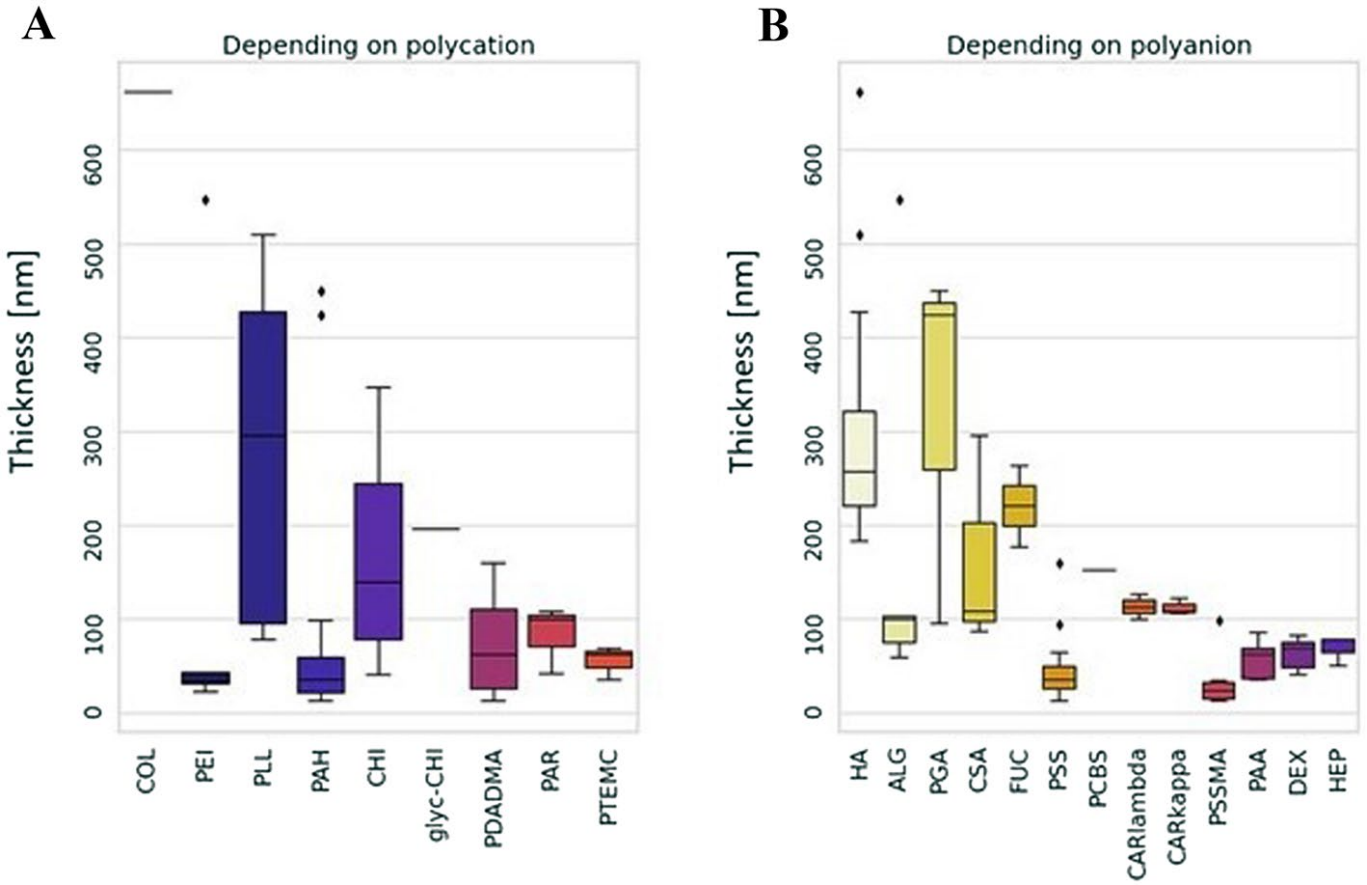
Boxplots showing the distribution of the thickness values in the coatings made of different polymers. (**A**) Thickness depending on polycations. (**B**) Thickness depending on polyanions. COL: collagen, PEI: polyethylenimine, PLL: poly(L-lysine), PAH: poly(allylamine hydrochloride), CHI: chitosan, glyc-CHI: glycol-chitosan, PDADMA: poly(diallyldimethylammonium chloride), PAR: poly(L-arginine), PTEMC: poly(trimethylammonium ethyl methacrylate chloride), HA: hyaluronic acid, ALG: alginate, PGA: poly(L-glutamic acid), CSA: chondroitin sulfate, FUC: fucoidan, PSS: poly(styrene sulfonate), PCBS: poly[1-[4-(3-carboxy-4-hydroxyphenylazo)benzenesulfonamido]-1,2-ethanediyl, sodium salt, CARlambda: λ-carrageenan, CARkappa: κ-carrageenan, PSSMA: poly(4-styrenesulfonic acid-co-maleic acid), PAA: poly(acrylic acid), DEX: dextran, HEP: heparin.

As a first step, distribution of the thickness values in the coatings made of different polymers was studied (Fig. 2). The results show that for polycations, the coatings made of poly(L-lysine) (PLL) have the greatest thickness median values with large interquartile range (IQR), which overlaps the thickness distribution of chitosan (CHI)-made coatings (Fig. 2A). In Fig. 2B, coatings having poly(L-glutamic acid) (PGA) have the greatest thickness median value with IQR overlapping with hyaluronic acid (HA)-containing coatings thickness distribution. The large thickness distribution of the LbL films containing the aforementioned polymers is probably due to the high frequency of their utilization and therefore to the wide variety of molecular weights (MW) that have been used.

**Figure 3:**
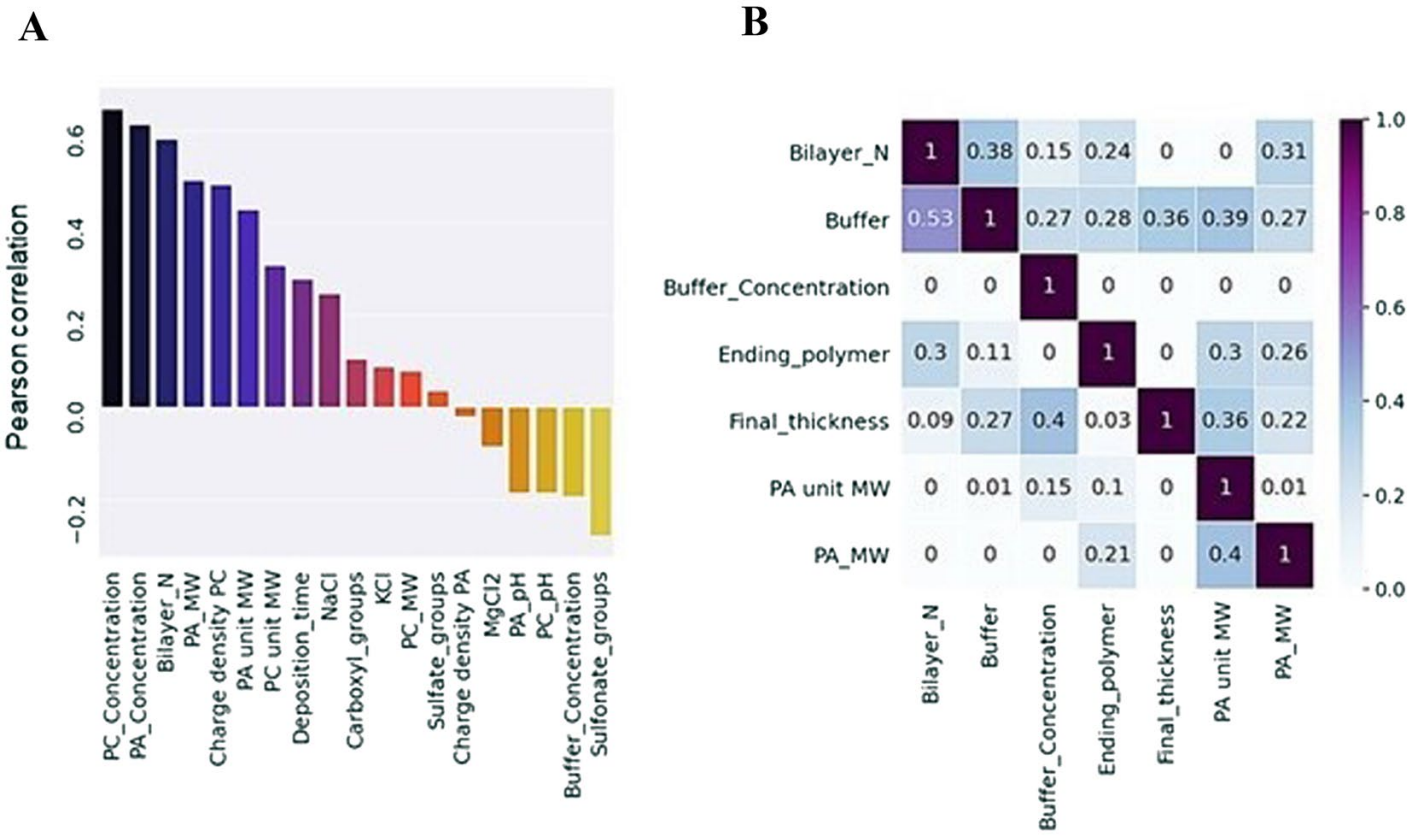
Pearson correlation (**A**) and Predictive Power Scores (PPS) (**B**) calculated for the final thickness of the coating and coating features. The first seven Pearson correlations are statistically significant (*p* ≤ 0.05). Only features having PPS > 0.001 with the target value are included. Full names of polymer features are provided in SI Table S3.

Next, linear relationships between the coating thickness and different parameters were evaluated using the Pearson correlation, and non-linear relationships were analyzed with the predictive power score (PPS) method (Fig. 3). The PPS allows seeing non-symmetrical relationships between variables: features located on the x-axis are independent variables (influencers), and features on the y-axis are dependent variables (influenced by x-features).

The results show that concentrations of polymers and the number of bilayers in the coating have a strong positive linear relationship with the resulting thickness (Fig. 3A), while polyanion molecular weight and buffer properties have some non-linear relationships with this property (Fig. 3B). The effect of concentration and the number of bilayers are intuitive, however, their relative importance in an overall tendency sense cannot be extracted from a single or few types of LbL films, which is the currently common practice. Similarly, the discrepancy between the relative importance of polyanion (PA) MW and polycation (PC) MW is also not evident.

One of the most important features which have strong linear relationships with the coating thickness is the number of bilayers in the coating (Fig. 3).

As stated above, this dependency is largely obvious, and therefore, the data had to be unified by this number. For this, we decided to calculate the thickness at eight bilayers for each coating in the literature-generated data using the growth function (Eq. 1).

The growth function, describing changes in coating thickness *d* with the number of layers, *N*, has three coefficients, *a*_0_, *a*, and *b*, which vary depending on the coatings^16,17^. In this function parameter, *b* defines function curvature: for *b* ≥ 0.05, the growth is exponential, for *b* < 0.05, it is nearly linear.

1 $$d = a_{0} + a*e^{bN}$$

We extracted data on the dynamics of each coating growth from the original research papers. Then we used these data to calculate coefficients of growth function. Having these coefficients, we defined the thickness of coatings having eight bilayers (N = 16). With this, we created a new target value, the thickness of the 8-bilayer coating, which was not dependent on the number of bilayers.

**Figure 4:**
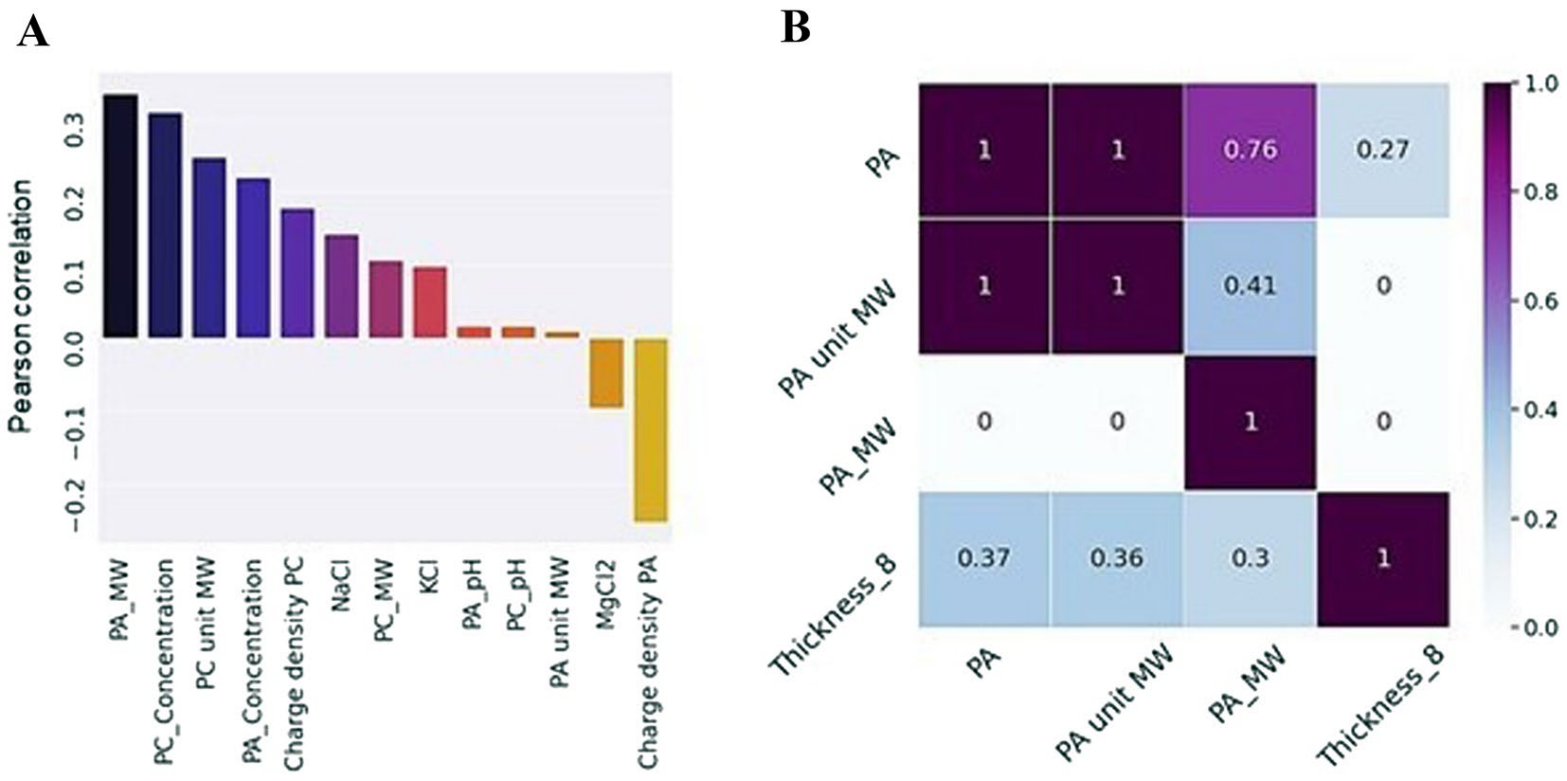
Pearson correlation (**A**) and Predictive Power Scores (PPS) (**B**) calculated for the thickness of the 8-bilayered coating and coating features. The first three and the last Pearson correlations are statistically significant (*p* ≤ 0.05). Only features having PPS > 0.001 with the target value are included. Full names of polymer features are provided in SI Table S3.

In this configuration, the type of polyanion, its concentration and its molecular weight were found to positively influence the thickness of the coating, while the charge density of polyanion negatively correlated with the coating thickness (Fig. 4A). Three features (on the x-axis) contributed to the coating thickness (on the y-axis) (Fig. 4B): type of polyanion, polyanion unit molecular weight, and resulting molecular weight. The reason why polyanion characteristics appear more important than those of polycation can be explained by pH values at which polyelectrolyte multilayers were built (pH values are close to the pKa of the acid groups).

### Coating thickness predictions

After the determination of the most influential parameters, the next step was the build-up of a predictive model. We constructed a Bagging Regressor model^18^ to make predictions about coating thickness using ten features from the original data set: presence of HA, presence of poly(styrene sulfonate) (PSS), resulting MW of the polycation and of the polyanion, unit MW of the polycation and of the polyanion, concentration of polycation and concentration of polyanion, the concentration of NaCl, and charge density of polyanion. Two quantitative structure–property relationship (QSPR) regression models were constructed using the selected features, Bagging Regressor and support vector regression (SVR) (Table 1).

**Table 1:** Performance of the constructed models measured as root-mean-square error (RMSE, nm) for three data sets. QSPR: quantitative structure–property relationship, SVR: support vector regression.

RMSE (nm)	QSPR Bagging regressor	QSPR SVR	Bagging regressor
Training set	46.7	76	50.9
Test set	68.7	113.8	73.8
Validation set	74.7	123.1	226.6

These models have smaller root-mean-square error (RMSE) values for the validation set than the Bagging Regressor constructed using the original dataset features. This fact indicates their greater potential for generalization. The model performance was evaluated with RMSE values calculated for the training set, test set, and validation set. All the models were fivefold cross-validated. All metrics are mean values for scores from different folds from cross-validation.

However, we encountered a classical Machine Learning challenge: overfitting, when the model makes good predictions for the instances it was built on (training set), but fails to “generalize”, i.e. make good predictions for the unseen items (validation and test sets). Therefore, the large gap between training and test/validation error values is the major sign of overfitting. This is the case for the Bagging Regressor constructed on the original data set features. As we can see, this model generates a large RMSE value for the validation set, which is 4 times larger than the error for the training set. From here, we conclude that polymers as specific chemical entities are not good features by themselves, and generating numerical features that describe the chemical properties of polymers can improve the model performance.

To get features of a molecule, firstly we had to get information about its structure, which is commonly represented in simplified molecular-input line-entry system (SMILES) format, available in the PubChem database. This information is further used to predict molecular features by deep learning pre-trained models available in the DeepChem library.

**Figure 5:**
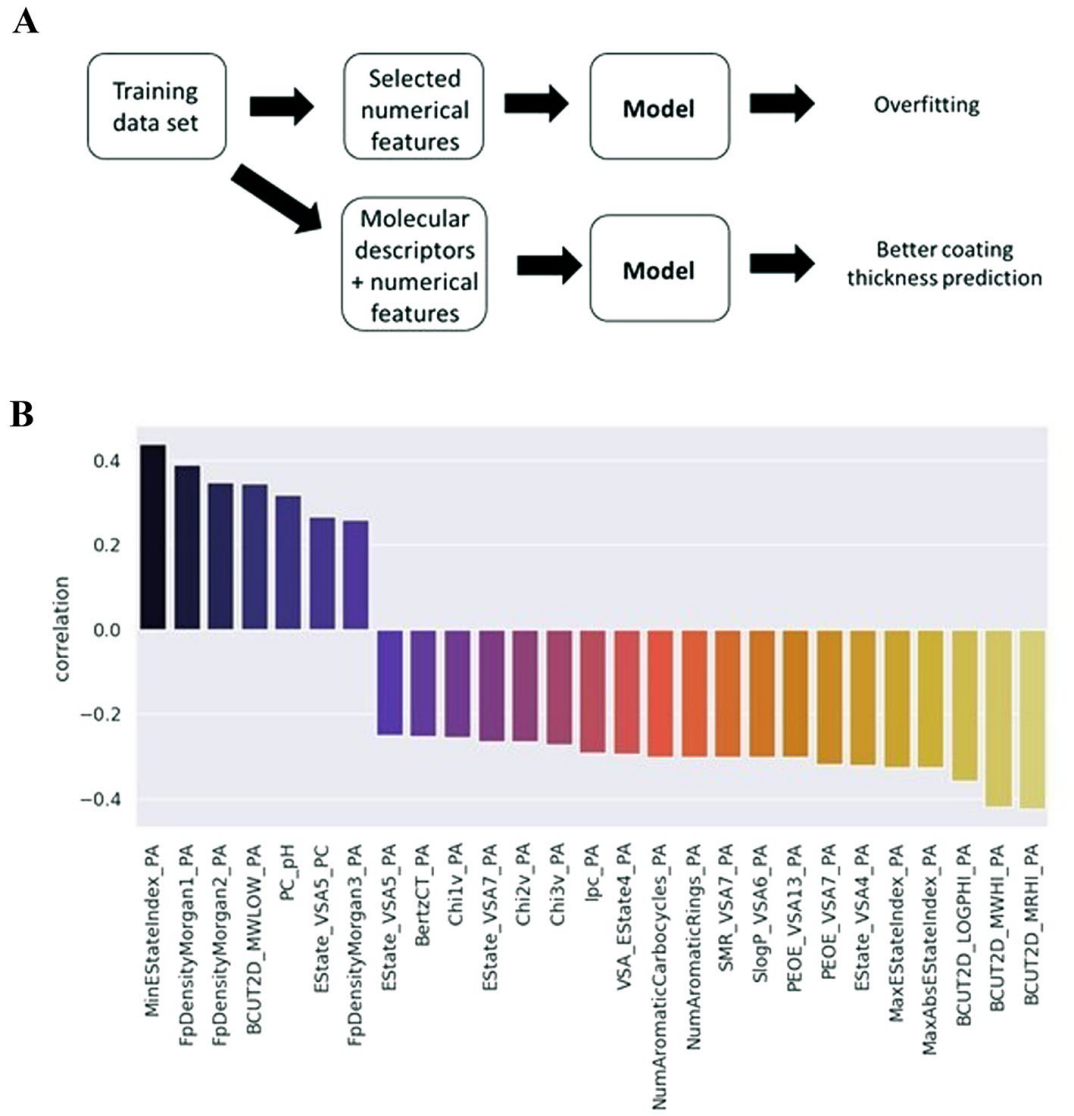
(**A**) Schematic presentation of the model building process. (**B**) Correlation between RDKit descriptors and thickness of the coating. Only descriptors with r ≥ 0.25 are shown. All coefficients are statistically significant (*p* ≤ 0.05).

For each polycation and polyanion, 123 molecular descriptors were generated, therefore each coating in the dataset was characterized by 246 molecular descriptors. Many molecular features have a moderate correlation with the thickness of the coating, so we assume that they can be used to predict this target value (Fig. 5). Mostly, molecular descriptors with high correlations with the coating thickness are related to polyanions, not polycations; this is in line with the observations in the previous section.

In the next step, we combined features generated by DeepChem and numerical features of the polymers from the original data set. After that, we performed feature elimination with the recursive feature elimination (RFE) algorithm leaving ten features that will be used by the models. These features are MW of the polycation, MW of the polyanion, NaCl concentration, six polyanion RDKit descriptors (MinAbsEStateIndex, FpDensityMorgan3, BalabanJ, PEOE_VSA8, VSA_EState2, VSA_EState6), and one polycation descriptor Kappa1. The most significant molecular descriptors demonstrate the importance of the topological features of the polyanions (such as Balaban distance connectivity index (BalabanJ) and also polycations (Kappa1) in the formation of the supramolecular LbL assemblies in addition to the electrostatic interactions which are described by molecular operation environment (MOE) type electrotopological descriptors. Descriptors related to van der Walls forces and also partial charges underline the highly intricate nature of the LbL film formation at molecular level.

As the last step, in order to further test the generalization capacity of the final model, we have tested polymers which were not in the training set that are described solely by molecular descriptors.

**Figure 6:**
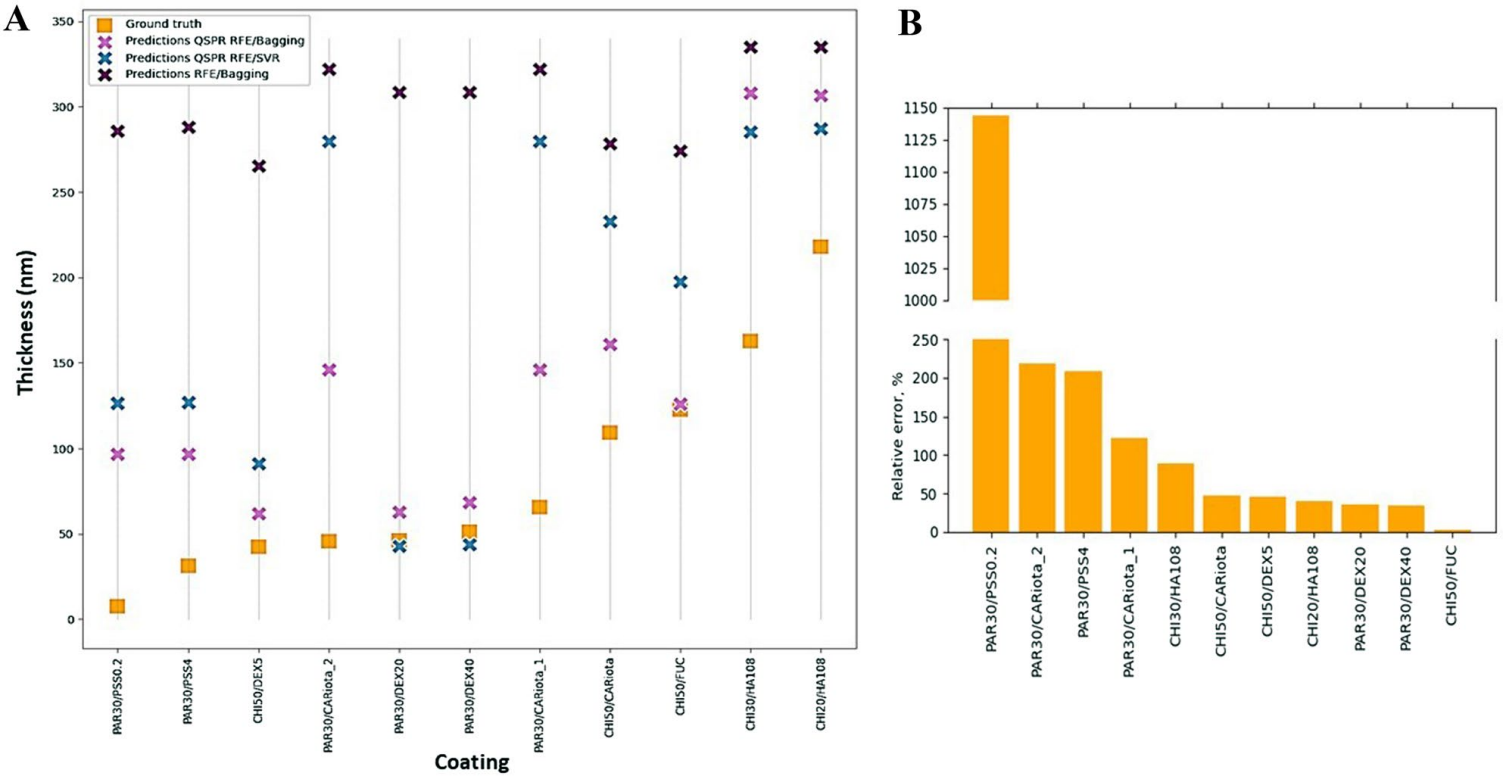
(**A**) Real (ground truth) and predicted thickness of coatings in the validation data set. (**B**) Relative errors for predictions made with the best of constructed models (QSPR RFE/Bagging).

In this configuration, we observed more inaccurate predictions (Table 2, Fig. 6). However, this could be expected, given the size of the training dataset. As we can see, the best results are obtained with the use of the QSPR RFE/Bagging regressor. It can predict the thickness of the coating better than two other models. We can also see that predictions of QSPR models are better than for the model RFE/Bagging constructed with original features, which predicts almost constant thickness values for all coatings (302 ± 25 nm). Predictions of QSPR models are correlating, and there are four coatings for which both regressors failed to predict thickness correctly (relative error > 100%): PAR30/PSS0.2, PAR30/CARiota_2, PAR30/PSS4, PAR30/CARiota_1 (Fig. 6B).

**Table 2:** The thickness of the coatings predicted with three models and thickness determined experimentally (ground truth).

Film	Ground truth, thickness (nm)	Predicted thickness, QSPR RFE/Bagging	Predicted thickness, QSPR RFE/SVR	Predicted thickness, RFE/Bagging
PAR30/DEX40	51.30	68.55	43.86	308.61
CHI50/DEX5	42.70	62.02	91.45	265.33
CHI50/FUC	123.00	125.84	197.79	274.23
CHI50/CARiota	109.30	161.07	232.98	278.50
PAR30/CARiota_1	65.80	146.22	279.71	322.28
PAR30/PSS0.2	7.80	97.00	126.70	285.69
PAR30/PSS4	31.50	97.00	126.89	288.20
PAR30/DEX20	46.00	62.74	42.77	308.61
CHI30/HA108	162.80	308.23	285.14	335.13
CHI20/HA108	218.20	306.78	287.21	335.13
PAR30/CARiota_2	45.90	146.22	279.71	322.28

Two other coatings, that have inaccurate predictions, contain CARiota polymer which was not in the dataset during training. The fact that the model fails to make predictions for the coatings which are made of unseen polymers confirms the assumption the generalization is not complete. It is interesting that despite this, there is one coating with CARiota for which relative error is small (47%), and the model was able to make an accurate prediction in this case.

Below, we discuss some of the possible reasons that may have caused large errors in the predictions made by the best of the constructed models, QSPR RFE/Bagging. For the PAR30/PSS0.2 and PAR30/PSS4, a large error may be caused by the too low molecular weight of the PSS polyanion compared to the one that the model has seen in the training set. The smallest polyanion in the dataset used for training was DEX with MW = 7.2 kDa, hereas in the validation set we have coatings where PSS has lower MW values (0.2 and 4 kDa). Moreover, in the training set, PSS MW was greater than in the validation set (60–70 kDa), so the combination of polyanion/MW of polyanion is new to the model. Because the model has made inaccurate predictions for the kind of polymers that it did not see during training, we assume that it can not generalize well and this is the reason for the prediction failures.

In the end, we observe that the model makes acceptable predictions for the coatings made of combinations of polymers that were present in the data set (like CHI/FUC, CHI/HA, and PAR/DEX). However, it fails to do so for the unseen polymers tested, due to the lack of extensive training data. We believe that the model potentially can be improved by generating and using more data for training. More data points will cover more chemical parameters, and the dependencies between features and thickness will be more informative and will have more predicting power.

## Conclusions and perspectives

In this work, we analyzed how different parameters such as polymer molecular weight, concentration, etc. affect LbL film thickness. After the determination of the most influential parameters, we used machine learning approach to verify if we can predict coating thickness from different parameters. We found that construction parameters alone were insufficient to build a robust model for thickness prediction because of the overfitting. To overcome this problem, we hypothetized that generating numerical features that describe the chemical properties of polymers can improve the model performance. Thus, we analysed the relationship between 123 molecular descriptors and the coating thickness, and found that molecular features had a moderate correlation with the thickness of the coating. Finally, we combined molecular descriptors and numerical features of the polymers from the original data set to build new models, and these models had better performance for the validation set than the model constructed using the original dataset features, which indicates their greater potential for generalization.

In conclusion, the generalization capacities of an algorithmic model predicting coating thickness can be improved by delving into the determining properties of the polymers in the context of LbL film formation dynamics. Harnessing the available data science techniques in biomaterial design and development such as multifunctional coatings will decrease lead time, empirical experimental load and also establish relationships between structure and function, which are otherwise hard to guess or estimate. The ultimate goal is to be able to predict coating functionalities based on polymer structure and buildup conditions, to develop innovative coatings for different applications.

## Materials and methods

### Materials

Alginate (ALG), λ-carrageenan (CARlambda), κ-carrageenan (CARkappa), ι-carrageenan (CARiota), chitosan (Mw = 50 and 100 kDa; CHI50 and CHI100), chondroitin sulfate (CSA), dextran (Mw = 5, 7.2, 20, 40 and 500 kDa; denoted respectively DEX5, DEX7, DEX20, DEX40 and DEX500), fucoidan (FUC), heparin (HEP), poly (styrene sulfonate) (0.2, 4.2, 15, 29, 70, 80, 145 and 2070 kDa; denoted respectively PSS02, PSS4, PSS15, PSS29, PSS70, PSS80, PSS145, PSS2600) were purchased from Sigma Aldrich, France. Chitosan (Mw = 20, 30 and 250 kDa; CHI20, CHI30 and CHI250) were purchased from Glentham Life Sciences, United Kingdom. Hyaluronic acid (Mw = 29, 108, 823 and 2670 kDa, HA29, HA108, HA823 and HA2670) were purchased from Lifecore Biomedical, USA. Poly(L-arginine) with 30 residues of arginine (PAR30) was purchased from Alamanda Polymers, USA.

### Data collection

To collect the data from the literature, we searched articles describing LbL film buildup using PubMed website and Google Scholar (keywords: LbL, polyelectrolyte, film, thickness). In total, 31 articles were found. Among them, only articles specifying the parameters of the film buildup, as well as film thickness, in the text and/or in the figures, were selected. Thus, 19 articles were used in this study^16,19–36^.

**Figure 7:**
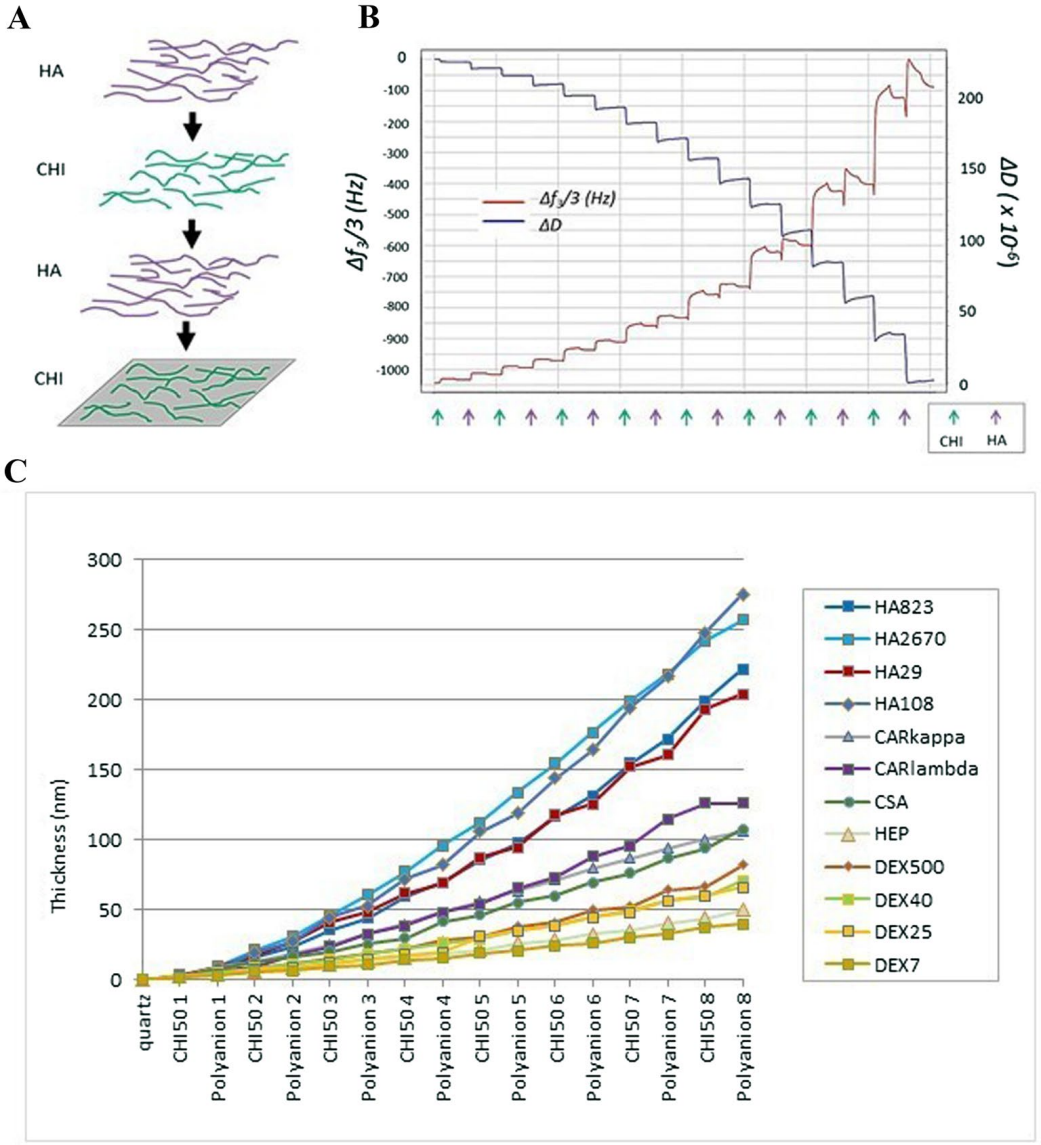
Film thickness determination by QCM-D. (**A**) Polyelectrolytes (chitosan, CHI, and hyaluronic acid, HA) are deposited on the crystal, where they form a multilayer film. (**B**) Growth of the film is followed by the measurements of the frequency and the dissipation variations with respect to the crystal in contact with the buffer solution only. As an example, the figure shows the measurements corresponding to the third overtone. (**C**) Film growth depending on polyanion.

The second dataset consisted of experimental data produced in the laboratory using QCM (Q-Sense, Sweden). For this, a SiO_2_ coated-crystal was excited at different resonance frequencies (fundamental frequency, third, fifth, and seventh overtones), and changes in frequency and dissipation were measured during the successive deposition of polymers and rinsing steps (Fig. 7A).

Before each experiment, the crystal was cleaned for 30 min with 2% Hellmanex, then rinsed with water. The final cleaning was done with 1 M HCl for 10 min, then rinsed with water. Poly(L-arginine) with chains composed of 30 arginine units (PAR30) and chitosan with different molecule weights (20, 30, 50, 100 and 250 kDa) (respectively CHI20, CHI30, CHI50, CHI100 and CHI250) were used as polycations. For the films constructed with PAR30 as polycation, a Tris 10 mM/NaCl 0.15 M at pH 7.4 buffer was used for the preparation of solutions and for rinsing steps. For the films constructed using chitosan as polycation, a buffer consisting of sodium acetate 70 mM/NaCl 80 mM at pH5 was used. Buffers were filtered with 0.2-µm filters. Twenty-eight different polymers were used as polyanions (see Materials part). QCM-D experiments were performed as described previously^37^. Briefly, polyanions and polycations used for the experiment were prepared at 0.5 mg mL^−1^ in acetate or Tris/NaCl buffers, as explained above. Polycations were first adsorbed to the surface for 5 min. A rinsing step was performed using the buffer for 5 min after each polyelectrolyte deposition, and 8 bilayers were constructed on the SiO_2_-coated crystal with a flow rate of 250 µL min^−1^.

Sauerbrey's equation gives the relation between the mass deposited on the vibrating crystal per unit area and the change of resonance frequency when the deposit behaves like a stiff coating of the crystal, i.e. when the deposit changes only the mass of the crystal. This is, however, generally not the case when a polyelectrolyte film is built up by successive depositions of polycations and polyanions from solutions. Indeed, on the one hand, the film is a viscoelastic body and on the other hand, the film is in contact with the solution. For both reasons, not only the change in frequency is measured but also the dissipation related to the characteristic damping time of the crystal vibration (Fig. 7B). If the normalized frequency shifts, Δ*f*_ν_/ν, corresponding to different overtone numbers are equal to − *m*/*C*, i.e. are independent of ν, Sauerbrey's equation stays valid. If this is not the case, a more sophisticated formalism ought to be used^38^. In this approach, the film is characterized by its elasticity, µ, its viscosity, η, its density, ρ, and its thickness, *d*. In the present study, the frequency shift and the dissipation corresponding to the overtones ν = 3, 5 and 7, i.e. for excitation frequencies of about 15, 25 and 35 MHz, have been processed to extract the thickness of the film at the end of the build-up (examples of film growth are shown in Fig. 7C). Hereafter, all thickness results are given for ρ = 1 g cm^−3^.

### Data analysis

The data set (Table S1), used for analysis and model construction, had 76 data points which were obtained by literature search (43 points) and experiments (33 points). The validation set used to evaluate model performance had 11 points, all generated by experiments (Table S2).

In the original data set, each data point represents one coating with 23 features and one target value (resulting coating thickness). The features are the type of polycation (PC) and type of polyanion (PA), type of ending polymer, PC unit molecular weight (MW) and PA unit MW, total PC and PA MWs, the concentration of PC and the concentration of PA, polycation pH and polyanion pH, the charge density of PC and charge density of PA, presence of carboxyl groups, presence of sulfonate groups, presence of sulfate groups, crosslinking method, bilayer deposition time, type of buffer, buffer concentration, the concentration of KCl, the concentration of MgCl_2_, and the concentration of NaCl (Table S3).

Relationships between features and target value were evaluated with the Pearson correlation coefficient and Predictive Power Score^39^. Statistical significance of the Pearson correlation coefficient was checked using confidence intervals for *p* = 0.05 after Fisher’s Z-transformation.

Three regression models were built to predict coating thickness: one based on features from the original data set, and two Quantitative Structure–Property Relationships (QSPR-type) models based on the RDKit molecular descriptors. To build QSPR models, firstly the SMILES (simplified molecular-input line-entry system) representations of molecules were obtained from the PubChem database. Then, for each polymer SMILES, 123 molecular descriptors were generated using RDKit Descriptors from DeepChem library^40^.

Features, that are further used to build models, were selected from original feature space and from molecular descriptors space with the Recursive Feature Elimination method having Random Forest as the basis. Finally, two methods were used to construct three coating thickness prediction models: Bagging Regression, QSPR Bagging Regression, and QSPR Support Vector Regression (SVR). All the described methods are implemented in the scikit-learn library^22^.

## Supplementary Information

Supplementary Legends.

Supplementary Table 1.

Supplementary Table 2.

Supplementary Table 3.
