# Supplementary material for: Prediction of coating thickness for polyelectrolyte multilayers via machine learning
Source: Sci Rep. 2021 Sep 21;11:18702. doi: 10.1038/s41598-021-98170-x (PMC8455527; doi:10.1038/s41598-021-98170-x)
Supplement: Supplementary file 1 — Supplementary Legends. [file 41598_2021_98170_MOESM1_ESM.docx]

**Prediction of coating thickness for polyelectrolyte multilayers via machine learning**

**Varvara Gribova, Anastasiia Navalikhina, Oleksandr Lysenko, Cynthia Calligaro, Eloïse Lebaudy, Lucie Deiber, Bernard Senger, Philippe Lavalle, Nihal Engin Vrana**

**Table Legends**

**Table S1.** Existing Literature data on coating thickness and the data generated for the study with coating parameter and thickness measurement method

**Table S2**. Coatings produced for model validation and coating parameters

**Table S3.** Abbreviations of polymer features
