# Supplementary material for: Prediction of coating thickness for polyelectrolyte multilayers via machine learning
Source: Sci Rep. 2021 Sep 21;11:18702. doi: 10.1038/s41598-021-98170-x (PMC8455527; doi:10.1038/s41598-021-98170-x)
Supplement: Supplementary file 4 — Supplementary Table 3. [file 41598_2021_98170_MOESM4_ESM.docx]

**Prediction of coating thickness for polyelectrolyte multilayers via machine learning**

**Varvara Gribova, Anastasiia Navalikhina, Oleksandr Lysenko, Cynthia Calligaro, Eloïse Lebaudy, Lucie Deiber, Bernard Senger, Philippe Lavalle, Nihal Engin Vrana**

**Table S3.** Abbreviations of polymer features.

| **Parameter** | **Abbreviation** |
| --- | --- |
| Polycation | PC |
| Polyanion | PA |
| Polycation MW, kDa | PC_M |
| Polyanion MW, kDa | PA_MW |
| Ending polymer | Ending_polymer |
| Concentration of polyanion, mg/mL | PA_Concentration |
| Concentration of polycation, mg/mL | PC_Concentration |
| Duration of each layer deposition, min | Deposition_time |
| Presence of negatively charged groups : COO^-^ (Y/N) | Carboxyl_groups |
| Presence of negatively charged groups sulfonates: -SO_3_ (Y/N) | Sulfonate_groups |
| Presence of negatively charged groups sulfates: -O-SO_3_ (Y/N) | Sulfate_groups |
| pH polyanion | PA_pH |
| pH polycation | PC_pH |
| Buffer concentration, mM | Buffer_Concentration |
| NaCl, M | NaCl |
| KCl, M | KCl |
| MgCl2, M | MgCl2 |
| Number of the bilayer | Bilayer_N |
| Film thickness, nm | Final_thickness |
| Charge density (charges per unit), polyanion | Charge density PA |
| Charge density (charges per unit), polycation | Charge density PC |
| Polycation unit MW, kDa | PC unit MW |
| Polyanion unit MW, kDa | PA unit MW |
